# A novel *de novo* frameshift deletion in *EHMT1* in a patient with Kleefstra Syndrome results in decreased H3K9 dimethylation

**DOI:** 10.1002/mgg3.268

**Published:** 2017-01-26

**Authors:** Patrick R. Blackburn, Monique Williams, Margot A. Cousin, Nicole J. Boczek, Geoffrey J. Beek, Gwen A. Lomberk, Raul A. Urrutia, Dusica Babovic‐Vuksanovic, Eric W. Klee

**Affiliations:** ^1^ Center for Individualized MedicineMayo ClinicJacksonvilleFlorida32224; ^2^ Department of Health Science ResearchMayo ClinicJacksonvilleFlorida32224; ^3^ Department of Biochemistry and Molecular BiologyMayo ClinicRochesterMinnesota55901; ^4^ Center for Individualized MedicineMayo ClinicRochesterMinnesota55901; ^5^ Department of Health Science ResearchMayo ClinicRochesterMinnesota55901; ^6^ Department of Clinical GenomicsMayo ClinicRochesterMinnesota55901; ^7^ Laboratory of Epigenetics and Chromatin DynamicsMayo ClinicRochesterMinnesota55901; ^8^ Division of Gastroenterology and HepatologyMayo ClinicRochesterMinnesota55901; ^9^ Department of Laboratory Medicine and PathologyMayo ClinicRochesterMinnesota55901

**Keywords:** EHMT1, functional validation, GLP, Kleefstra Syndrome, p.Arg310Aspfs*4, whole exome sequencing

## Abstract

**Background:**

Kleefstra Syndrome (KS) (MIM# 610253) is an autosomal dominant disorder caused by haploinsufficiency of euchromatic histone methyltransferase‐1 (*EHMT1,* GLP). *EHMT1* (MIM# 607001) encodes a histone methyltransferase that heterodimerizes with EHMT2 (also known as G9a, MIM# 604599), which together are responsible for mono‐ and dimethylation of H3 lysine 9 (H3K9me1 and ‐me2), resulting in transcriptional repression of target genes.

**Methods:**

This report describes an 18‐year‐old woman with intellectual disability, severely limited speech, hypotonia, microcephaly, and facial dysmorphisms, who was found to have a novel *de novo* single‐base frameshift deletion in *EHMT1*.

**Results:**

Functional studies using patient fibroblasts showed decreased H3K9me2 compared to wild‐type control cells, thus providing a rapid confirmatory test that complements molecular studies.

**Conclusion:**

Whole exome sequencing revealed a novel frameshift deletion in *EHMT1* after a lengthy diagnostic odyssey in this patient. Functional testing using this patient's fibroblasts provides proof‐of‐concept for the analysis of variants of uncertain significance that are predicted to impact EHMT1 enzymatic activity.

## Introduction

Kleefstra syndrome (KS) (MIM# 610253, also known as 9q subtelomeric deletion syndrome) is characterized by severe developmental delay, absent or limited speech, hypotonia, brachycephaly, distinctive facial features, congenital heart defects, and behavioral problems (Kleefstra et al. 2006). Studies of individuals with 9q subtelomeric deletions showed that the euchromatic histone methyltransferase‐1 gene (*EHMT1,* MIM# 607001) falls within the critical region deleted in these patients, and further investigations revealed small interstitial deletions affecting *EHMT1* (Kleefstra et al. 2006). Subsequent studies on additional individuals with intragenic mutations in *EHMT1*, including nonsense, splice site, and missense variants, confirmed that haploinsufficiency of *EHMT1* results in KS (Kleefstra et al. 2009).

The exact mechanism by which haploinsufficiency of *EHMT1* leads to KS in patients is not known. *Ehmt1* ^*+/−*^ heterozygous knockout mice show abnormal social behavior with measurable deficits in spatial learning and memory that are associated with structural and functional synaptic defects, particularly within CA1 hippocampal neurons (Balemans et al. 2013). More recently, EHMT1 has been shown to be important in regulating homeostatic plasticity and is a critical regulator of synaptic scaling in response to changes in neuronal activity that can affect a range of developmental and adaptive cognitive processes (Benevento et al. 2016). Haploinsufficiency of *EHMT1* is predicted to disrupt homeostatic plasticity, which may lead to improper neural circuit formation during development in patients with KS (Benevento et al. 2016).

In this study, we describe an 18‐year‐old woman with global developmental delay, severely limited speech, hypotonia, microcephaly, and facial dysmorphisms, who was found to have a novel *de novo* single‐base frameshift deletion in *EHMT1* (Chr9(GRCh37): g.140637927_140637928del; NM_024757.4(EHMT1): c.928_929del; NP_079033.4: p.Arg310Aspfs*4) by whole exome sequencing (WES) after a protracted diagnostic odyssey. While the patient's phenotype is consistent with a diagnosis of KS, only ~100 cases have been described to date in the literature and this case contributes yet another novel variant to the growing body of literature surrounding this rare genetic disorder. This mutation was predicted to result in a premature truncation of the EHMT1 protein, and functional studies on the patient's fibroblasts demonstrated a decrease in H3K9me2 compared to wild‐type control cells and confirmed a lack of compensation in global H3K9me2 levels by EHMT2 (MIM# 604599). These functional assays provide a rapid readout of global *EHMT1* enzymatic activity and may be useful in other cases of suspected KS with missense variants of unknown significance (VUS) that are predicted to impact *EHMT1* function.

### Clinical report

The patient was a 6 lbs. 5 oz. child born to a 39‐year‐old mother following uncomplicated pregnancy with no reported fetal exposures and is the mother's first and only child. Delivery was by induced labor at 39 weeks, with some fetal distress and Apgars of eight at 1 min and nine at 5 min after birth. The patient received phototherapy for hyperbilirubinemia (maximum bilirubin of 15.1 mg/dL) for 10 days and intravenous glucose for profound hypoglycemia (glucose level of 14 and 18 mg/dL) at 48 h after birth. The patient developed chronic constipation beginning at 8 months with occasional acute abdominal distension with evidence of obstruction. Abdominal ultrasound was normal with no evidence of organomegaly, and the liver, kidneys, and bladder were all normal. The patient was sitting at 8 months but was hypotonic, with very unstable sitting balance, and walked with assistance on her tip‐toes at ~15 months. During her initial workup at 6 months, the patient was noted to have microbrachycephaly, ear crease, widely spaced eyes, epicanthal folds, upslanted palpebral fissures, mild intermittent esotropia, umbilical hernia, and heart murmur. Further evaluation of her heart murmur by two‐dimensional echocardiography was normal. The patient followed the 75–90th percentile for height and weight but had microcephaly since early childhood. Her head circumference was 46.2 cm at 2 years and 11 months (6th percentile, −1.6 SD) and 47.5 cm by 5 years and 5 months (1st percentile, −2.2 SD). Electroencephalography (EEG) studies were normal. The patient never developed speech (language functioning at the 18–24 month level) and currently relies on her iPad for communication. Psychometric evaluation showed that the patient scored very low with an IQ in the range of low 40s. The patient was diagnosed with autism and associated developmental delays, and exhibits atypical behaviors including inattentiveness, anxiety, fearfulness, preoccupation, and stereotypical motor behaviors.

### Laboratory testing

Initially, the patient was evaluated for Down syndrome (MIM# 190685), but chromosomal analysis from peripheral blood showed normal 46, XX karyotype at 475 bands (Mayo Medical Laboratories). Based on her facial appearance, which was somewhat coarse with synophrys, the possibility of Cornelia de Lange syndrome (MIM# 122470) was raised and *NIPBL* (MIM# 608667) gene testing was performed but did not reveal any abnormalities (University of Chicago Genetic Services Laboratories). A blood sample was sent for microarray studies (Kleberg Cytogenetics Laboratory at the Baylor College of Medicine) and reported a duplication of two clones (RP11‐1331H7 and GS‐820M16) in the telomeric region of chromosome 14, which could not be confirmed by FISH analysis. Paternal testing revealed that this duplication was inherited from the patient's father, who was phenotypically unremarkable. Clinical microarray testing was repeated locally and revealed an interstitial duplication at 6q11.1(62,025,212–62,958,462) [hg18], spanning 933 kb (Mayo Medical Laboratories). This duplication includes a single gene, *KHDRBS2* (MIM# 610487), which has not been found to be associated with human disease. FISH testing (RP11‐1142N18) of a paternal sample also demonstrated that this duplication was inherited from the father and is most likely a familial variant without phenotypic significance.

Due to developmental delay associated with neonatal hypoglycemia, microcephaly, large tongue, and a skeletal survey at 16 months that showed advanced bone age (close to the 24 month standard; >2 standard deviations from the mean) the patient was evaluated for Beckwith‐Wiedemann syndrome (MIM# 130650). The patient had negative uniparental disomy (UPD) studies for chromosome 11 and was negative for *KCNQ1OT1* (*LIT1*, MIM# 604115) hypomethylation (index was 0.48; Normal index range 0.41–0.59). The patient had a number of negative cytogenetic studies, including FISH to investigate 15q11.2 and 22q11.2 chromosomal regions, and FISH for Smith–Magenis syndrome (MIM# 182290). She also had normal FISH using telomere probes. Molecular testing for Prader–Willi (MIM# 176270) and Angelman syndrome (MIM# 105830) was negative (methylation studies and FISH using SNRPN and D15S10 probes) and *UBE3A* (MIM# 601623) gene sequencing was also normal. The patient had normal molecular testing for Rett syndrome (MIM# 312750, *MECP2* gene, MIM# 300005) and normal molecular testing for Fragile X syndrome (MIM# 300624, *FMR1* gene, MIM# 309550). She was screened for Smith–Lemli–Opitz syndrome (MIM# 270400), and this was normal. The patient also had a negative biochemical screen that included urine organic acids, purine/pyrimidine panel, as well as plasma lactate, ammonia, uric acid, carnitine, and acylcarnitine profiles.

### MRI evaluation

Magnetic resonance imaging (MRI) showed hypoplasia of the cerebellar vermis and the superior cerebellar peduncles were smaller than expected. Scattered areas of high signal in the periatrial white matter, and subcortical white matter of the frontal, parietal, and occipital lobes was observed, but was largely nonspecific and thought to represent areas of delayed myelination. Based on these findings, the patient had fibroblast testing to determine levels of alpha‐mannosidase (alpha‐mannosidosis, types I and II, MIM# 248500), arylsulfatase‐A (metachromatic leukodystrophy, MIM# 250100), beta‐galactosidase (GM1‐gangliosidosis, MIM# 230500 and mucopolysaccharidosis type IVB (Morquio), MIM# 253010), beta‐glucuronidase (mucopolysaccharidosis VII, MIM# 253220), cerebroside beta‐galactosidase (Krabbe disease, MIM# 245200), and hexosaminidase (Tay–Sachs disease, MIM# 272800), which were normal, thus excluding these disorders.

## Methods

### Ethical compliance

The patient or their parents consented for sample collection and subsequent analysis under a protocol approved by the institutional review board of the Mayo Clinic and consent was obtained to publish patient photographs.

### Whole exome sequencing

Clinical whole exome sequencing was performed at Baylor Miraca Genetics. The laboratory performs massively parallel sequencing on an Illumina HiSeq with 100 base paired‐end reads. Generally, the following quality control metrics are achieved: >70% of reads aligned to target, 95% of target bases covered at >20×, 85% of target bases covered at >40×, and mean coverage of the target bases is >100×.

### Cell culture and maintenance

Normal and *EHMT1* ^+/−^ mutant human dermal fibroblasts were cultured and maintained in Minimum Essential Medium (Invitrogen, Grand Island, NY, USA) supplemented with 10% fetal bovine serum, 0.1% Antibiotic/Antimycotic (Invitrogen, Grand Island, NY, USA), and 0.1% MEM Nonessential amino acids (Corning Inc., Manassas, VA, USA) at 37 ^⁰^C with 5% CO_2_.

### Immunofluorescence analysis of H3K9me2 in control and patient fibroblasts

Normal and *EHMT1* ^+/−^ mutant human dermal fibroblasts were plated on polylysine‐coated circular coverslips (0.1 mg/mL polylysine) and allowed to adhere overnight. Cells were then fixed with 4% formaldehyde, permeabilized with 0.2% Triton‐X 100, blocked for 30 min, stained with H3K9me2 (Cell Signaling Technology, Danvers, MA, USA) primary and Alexa Fluor 488 secondary antibody (Invitrogen, Grand Island, NY, USA). Coverslips were mounted in VectaShield mounting media (with DAPI) for immunofluorescence. Images were acquired using 40× and 100× objective lenses on a Zeiss LSM 780 confocal microscope.

### Western blot analysis

Normal and *EHMT1* ^+/‐^ mutant human dermal fibroblasts were lysed in 4× Laemmli buffer (250 mm Tris (pH 6.8), 20% glycerol, 8% SDS, 0.0025% bromophenol blue, 1 mm *β*‐mercaptoethanol), collected by scraping with a rubber policeman and then sonicated for 10s. Samples were boiled at 95 ^⁰^C for 5 min then subjected to 14% SDS polyacrylamide gel electrophoresis, transferred onto polyvinylidene difluoride membranes and probed with the following primary antibodies: anti‐H3K9me2 (Cell Signaling Technology, Danvers, MA, USA), anti‐total Histone H3 (Abcam, Cambridge, MA, USA) in 3% bovine serum albumin in TBST, or anti‐EHMT1 (Abcam, Cambridge, MA, USA) in 5% milk. Anti‐mouse or anti‐rabbit secondary antibodies (Millipore, Danvers, MA, USA) were incubated on the membranes, after three successive washes with TBST, for 1 h at room temperature, followed by detection of bands with ECL (Pierce, Rockford, IL, USA). Quantification of bands was done using ImageJ (Rasband).

## Results

The patient underwent whole exome sequencing at the age of 17 (Baylor Miraca Genetics, Houston, TX, USA), which revealed a novel *de novo* single‐base frameshift deletion (Chr9(GRCh37): g.140637927_140637928del; NM_024757.4(EHMT1): c.928_929del; NP_079033.4: p.Arg310Aspfs*4) in the *EHMT1* gene that is implicated in KS. The *EHMT1* gene encodes a histone 3 lysine 9 (H3K9) methyltransferase that is necessary for the mono‐ and dimethylation of H3K9 (H3K9me1 and H3K9me2) in euchromatin that serves as a mark of transcriptional repression. This variant was classified as pathogenic by clinical report and according to 2015 ACMG guidelines (Richards et al. 2015). Four additional variants of uncertain significance (VUSs) were also reported, but were implicated in autosomal recessive conditions with minimal clinical overlap and were not felt to contribute to the patient's phenotype. The *EHMT1* c.928_929 deletion results in a frameshift and premature stop in the *EHMT1* transcript, which likely undergoes nonsense‐mediated decay.

**Figure 1:**
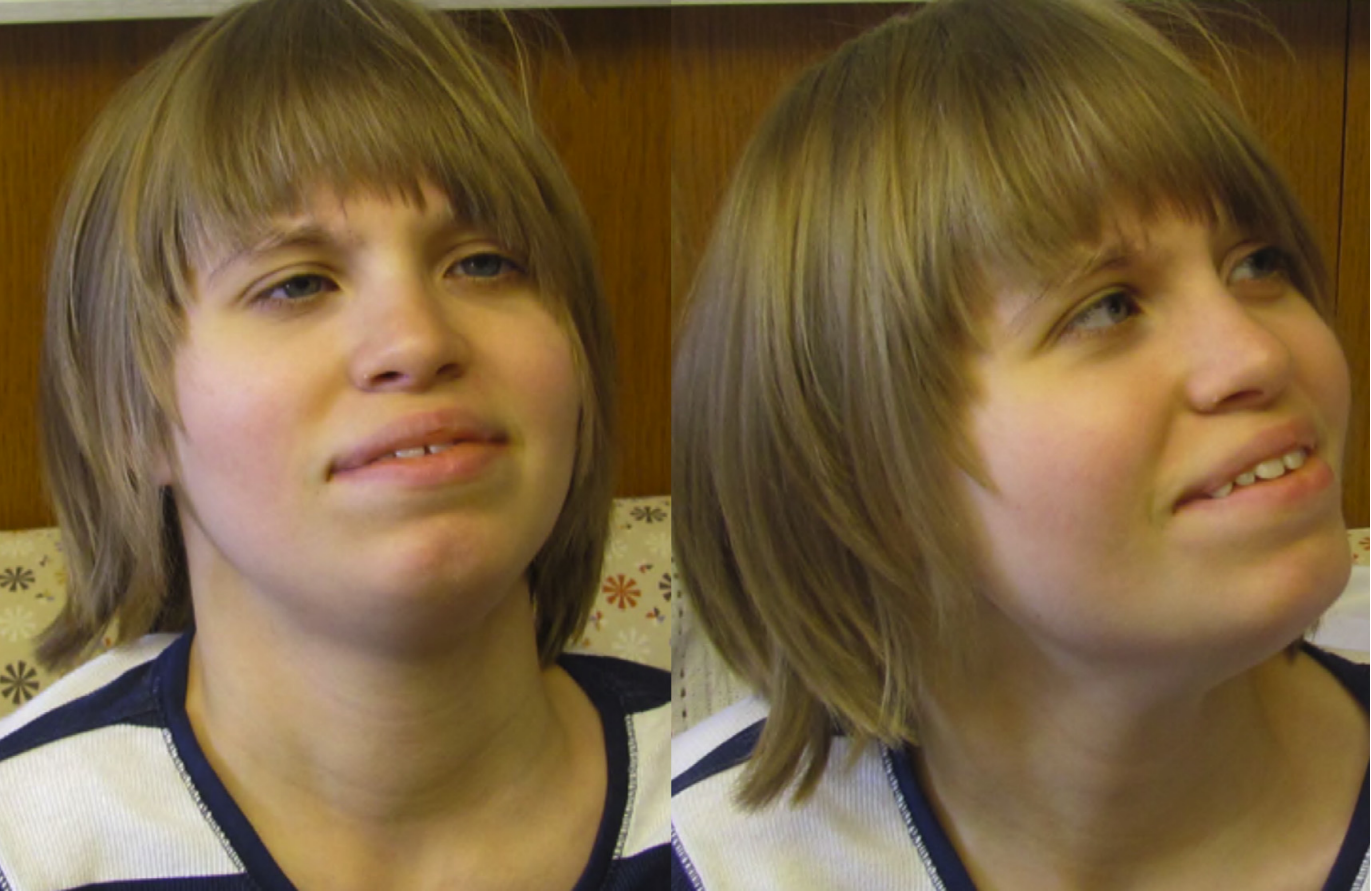
Patient photographs at 14 years of age showing features typical of Kleefstra syndrome. The patient has microcephaly, eyes widely spaced with epicanthal folds, ears that are overfolded and posteriorly rotated, with a deep notch on the left ear lobe (not shown), a short nose that is wide at the base, synophrys, and relative prognathism.

Given the extensive phenotypic overlap between our patient and previously reported cases of KS, it was felt that the pathogenic frameshift variant in *EHMT1* was the cause of disease in our patient. Our patient had global developmental delay, lack of speech development, hypotonia, microcephaly, abnormal behaviors, and facial dysmorphisms that are characteristic of this disorder (Fig. 1). She also had nonspecific white matter lesions and delays in myelination that were evident early in life. A recent study has suggested that reversible white matter lesions are a common clinical finding in KS and suggest that EHMT1 may be involved in myelination development, as the formation of myelinating oligodendrocytes requires H3K9 methylation (He et al. 2016).

**Figure 2:**
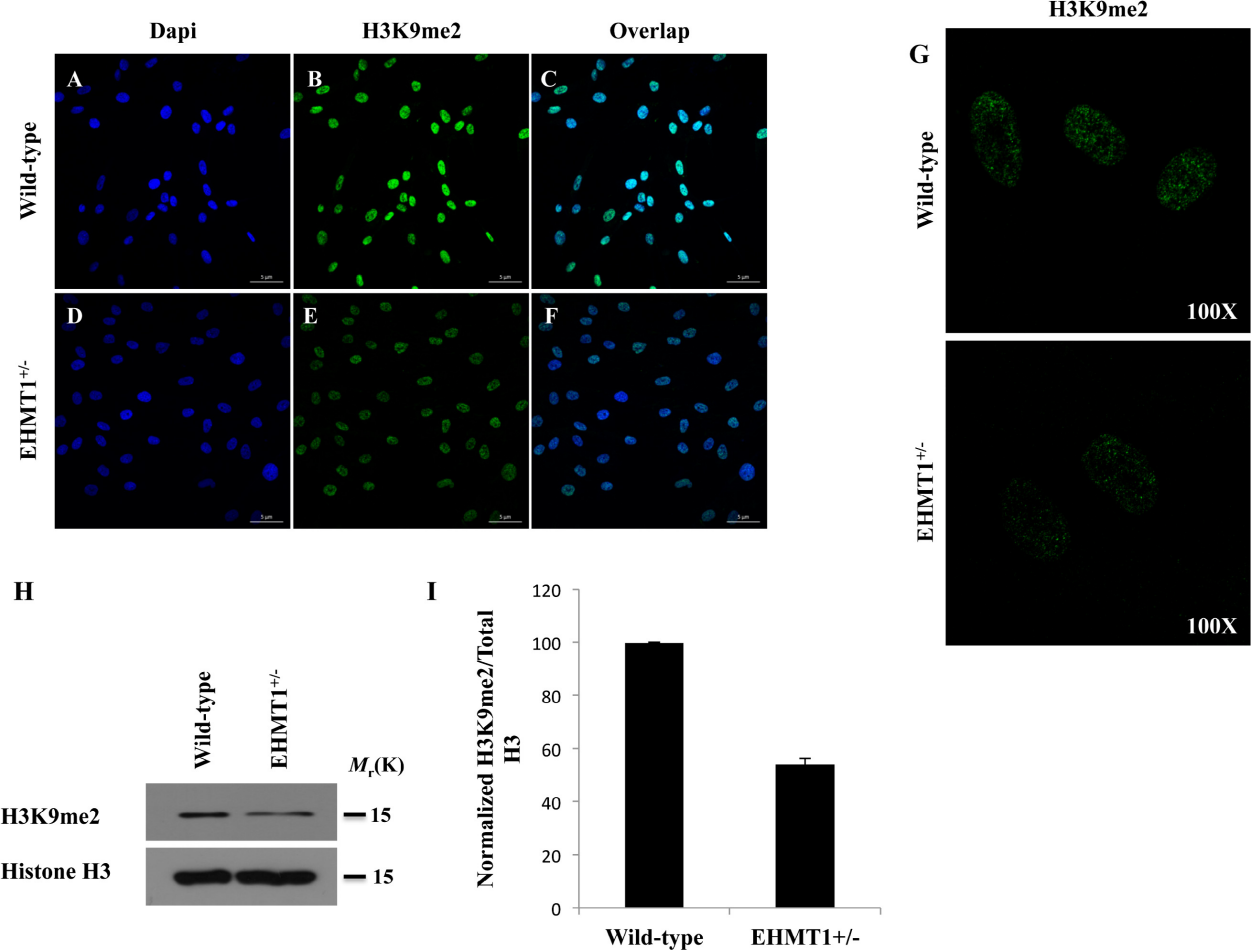
Novel patient *EHMT1* variant results in reduced H3K9 dimethylation. (A–F) Immunofluorescence microscopy analysis of H3K9me2 levels in wild‐type and EHMT1^+/−^‐mutant human fibroblasts illustrates a marked reduction in this histone mark in the mutant cells. (A, D) DAPI nuclear stain, (B, E) H3K9me2, (C, F) overlap of DAPI and H3K9me2. (H) Western blot analysis of total Histone H3 and H3K9me2 protein levels in the same cells confirms the reduction of H3K9me2 in the mutant cells relative to the wild‐type control. (I) Quantification of *n* = 3 western blots using ImageJ.

Since fibroblasts were available from previous clinical testing done on this patient, we decided to look at global H3K9me2 levels compared to wild‐type control fibroblasts to see whether haploinsufficiency of EHMT1 was compensated for in any way by EHMT2 (Fig. 2). Immunofluorescence analysis of H3K9me2 in patient and control fibroblasts showed a significant reduction in H3K9me2 within cell nuclei (Fig. 2A–F) as well as positive foci within the nucleus (Fig. 2G). H3K9me2‐specific antibodies were used to quantify total protein from cell lysates by western blot and showed approximately half as much H3K9me2 in patient cells compared to control cells when normalized to total H3 protein expression (54.22 ± 2.07% of control cells) (Fig. 2H,I). These findings were confirmed in a biological replicate, suggesting that H3K9me2 levels in patient fibroblasts could provide a biological readout for *EHMT1* haploinsufficiency and confirm the molecular findings for this patient.

## Discussion

Next‐generation sequencing is being increasingly utilized in the clinic and has the potential to reduce the time and expense associated with finding a clinical diagnosis in individuals affected by disorders with a putative genetic etiology. In this report, we describe a patient with a novel heterozygous *de novo* single‐base frameshift deletion in *EHMT1* with KS that was diagnosed using WES after a lengthy 17‐year diagnostic odyssey. The c.928_929 deletion was predicted to result in premature termination and loss of protein expression, resulting in haploinsufficiency of *EHMT1*. The patient had many of the classic phenotypic features associated with disease, and additional functional studies performed on patient fibroblasts showed a ~50% decrease in H3K9me2, a mark of transcriptional repression that is catalyzed by EHMT1/2 heterodimers. While functional testing was not essential in this case to diagnose the patient with KS, several recent studies have uncovered missense variants in *EHMT1,* which may contribute to disease or alternatively represent benign private mutations without any impact on EHMT1 function (Jiang et al. 2013; Nolan and Carlson 2016; Priest et al. 2016). This functional assay may be useful in unraveling the role of these novel missense variants and help guide future studies.
